# Multivariate multilevel modeling of quality of life dynamics of HIV infected patients

**DOI:** 10.1186/s12955-020-01330-2

**Published:** 2020-03-24

**Authors:** Zelalem G. Dessie, Temesgen Zewotir, Henry Mwambi, Delia North

**Affiliations:** 1 School of Mathematics, Statistics and Computer Science, University of KwaZulu-Natal, Durban, South Africa; 2 College of Science, Bahir Dar University, Bahir Dar, Ethiopia

**Keywords:** Correlation matrix, long term trend, Principal component, Latent variables

## Abstract

**Background:**

Longitudinal quality of life (QoL) is an important outcome in many chronic illness studies aiming to evaluate the efficiency of care both at the patient and health system level. Although many QoL studies involve multiple correlated hierarchical outcome measures, very few of them use multivariate modeling. In this work, we modeled the long-term dynamics of QoL scores accounting for the correlation between the QoL scores in a multilevel multivariate framework and to compare the effects of covariates across the outcomes.

**Methods:**

The data is from an ongoing prospective cohort study conducted amongst adult women who were HIV-infected and on the treatment in Kwazulu-Natal, South Africa. Independent and related QoL outcome multivariate multilevel models were presented and compared.

**Results:**

The analysis showed that related outcome multivariate multilevel models fit better for our data used. Our analyses also revealed that higher educational levels, middle age, stable sex partners and higher weights had a significant effect on better improvements in the rate of change of QoL scores of HIV infected patients. Similarly, patients without TB co-infection, without thrombocytopenia, with lower viral load, with higher CD4 cell count levels, with higher electrolytes component score, with higher red blood cell (RBC) component score and with lower liver abnormality component score, were associated with significantly improved the rate of change of QoL, amongst HIV infected patients.

**Conclusion:**

It is hoped that the article will help applied researchers to familiarize themselves with the models and including interpretation of results. Furthermore, three issues are highlighted: model building of multivariate multilevel outcomes, how this model can be used to assess multivariate assumptions, involving fixed effects (for example, to examine the size of the covariate effect varying across QoL domain scores) and random effects (for example, to examine the rate of change in one response variable associated to changes in the other).

## Background

Acquired immune deficiency syndrome (AIDS) affects not only the patients’ physical condition but also their mental health, financial aspects and social relationships [1]. However, the arrival of ART and its widespread availability in many settings has improved quality of life (QoL), suppressed viral loads and reduced the mortality rate among people living with HIV/AIDS [2]. Notably, QoL is predictive of good adherence to life-long therapy [3, 4] and is also an important marker of the well-being of patients living with a chronic disease for which there is currently no cure [5]. As the longevity of people living with HIV (PLWH) improves as a result of ART, assessment of QoL of the patient has become an important issue for policymakers and clinicians to plan for accessible, comprehensive and effective HIV care services [6]. It is also important to identify the factors affecting a patient’s QoL that are most amenable to intervention in this context.

QoL is a multi-dimensional and dynamic conception [7], including significant personal examinations on comprehensive aspects of patients well beings, physical health status, functional capacity, social-relationship, emotional wellbeing [8] and even spiritual well-being over time [9]. Herein, we define QoL as per world health organization (WHO) definitions as an individual’s awareness of their position in life in the context of culture and value systems in which they live and in relation to their expectations, goals, standards, and concerns [10]. According to WHO, QoL involves a wide-ranging assessment of the perception of an individual regarding the psychological, physical health, social-relationships, level of independence, environment and personal belief domains. Based on this, WHO QoL Brief version instrument was developed to capture multi-dimensional perspectives of QoL [11].

Several studies have employed linear regression for cross-sectional data [12–17] and linear mixed effects for longitudinal data [18–22] to evaluate the QoL of patients. These studies have assumed independence between QoL domain scores, estimating a separate model for each QoL domain score. However, independence is an unlikely assumption since health conditions may concurrently affect multiple aspects of life. For instance, physical health scores (i.e severe pain, fatigue and lack of energy) may be affected by psychological well-being scores (i.e. anxiety and depression). Furthermore, as noted [23], the residual correlation from such a univariate regression or linear mixed model shows the violation the conditional independence outcome. Consequently, the violation of this independence assumption can result in biased *p*-values, incorrect confidence intervals, and inflated effect sizes [24]. The multivariate multilevel methods play an important role to get rid of these kinds of restrictions by allowing variation at different levels and accounting for multivariate correlations [25].

Few researchers employ multivariate techniques to evaluate cross-sectional QoL outcomes. In particular, Conigliani et al. [26] and Hernández-Alava and Pudney [27] model EuroQoL *5-dimension* (*EQ-5D*) domain scores using multivariate ordered probit models and Copula ordinal regression, respectively. However, those papers were confined to the use of multivariate method for cross-sectional, discontinuous, non-normal and truncated data at both ends (i.e. the domain scores range from − 0.594 to 1). In our study, we extended the multivariate method in such a way that the correlations among the observations within a cluster (subject) and the dependence between the QoL domains scores are well accounted for. This is done by modelling the long-term trend of QoL domain scores based on multivariate multilevel models. In addition to that, the effect of several prognostic factors on the baseline and rate of change of QoL domain scores of HIV infected patients was investigated including white blood cell, red blood cell, and blood chemistry parameters. Another important case that we examined is whether the predictor variables have different relationships across QoL domain scores over time.

## Methods

### Study population

The data is from CAPRISA 002 Acute HIV infection study which is an ongoing prospective cohort study conducted among HIV-infected women. Patients were recruited at two sites in KwaZulu-Natal-South Africa, a rural site in Vulindlela and an urban site in the city of Durban. The original study, which started in 2004, enrolled a cohort of HIV uninfected women whose age was greater than 18 years with the aim to describe immunologic, clinical and virologic characteristics of HIV-1 disease [28]. Patients who seroconverted during the HIV uninfected stage were enrolled in the Acute HIV infection phase, and then followed-up during chronic infection, ART initiation, and up to 6 years on ART. Participants without well documented estimated date of HIV infection, and those who did not have at least two follow-up clinical attribute measurements, were excluded. Finally, two hundred and nineteen (219) participants were included in the study. Further information about the ongoing prospective HIV cohort study (CAPRISA-002), including women eligibility criteria and the enrollment procedure, were reported in the past studies [28, 29].

CAPRISA 002 trails initially enrolled HIV-negative (phase I) women into different study cohorts. The women who seroconverted were enrolled into the acute infection phase (i.e. phase II: weekly visits up to 3 months post-infection), then into early infection (i.e. phase III: monthly visits from 3 to 12 months) subsequently into established infection (i.e. phase IV: quarterly visits for more than 12 months) and afterward on cART (phase V). For the purpose of this study, samples for immunologic, virologic, QoL domain scores and clinical parameters were measured at each visit (i.e. from phase II to V). These longitudinal QoL outcomes, immunologic, virologic, and clinical measurements were measured for several followed-up visits. Of all the 9256 follow-up visits, we dropped 496 visits (5.4%) as patients did not have complete responses for QoL domain scores and clinical parameters, resulting a total of 8760 followed-up visits from 219 HIV infected women.

### Variables and measurements

Once enrolled in the study, face-to-face interviews were conducted in either isiZulu or English by trained counselors, nurses or research clinicians and study data were obtained at various stages depending on the types of measures being assessed. QoL was assessed using the World Health Organization quality of life (WHO-QoL) HIV BREF instrument [10]. All items are on a 5-point Likert scale, in which 1 indicates low and 5 indicates high perceptions. The average scores of all items in each domain were multiplied by 4 to convert domain scores to the range of 4 to 20 to make it comparable with the scoring pattern of WHO-QoL-100 [11]. For the purpose of this study, we used the following domains score. Firstly, the physical health scores, that measure the impact of the disease on the activities of daily living, dependence on therapeutic substances, presence of pain, fatigue, lack of energy and initiative and perceived working capacity. The second is about the psychological score domain that assesses the patient’s thoughts about body appearance, positive and negative feelings, self-esteem and personal beliefs, higher cognitive functions, spirituality, anxiety, suicide, and depression. The third domain is about social relationships which assesses personal relationships, social contacts, social support, and sexual activity. The fourth domain is devoted to the level of independence and assesses areas such as mobility, activities of daily living, dependence on treatments and work capacity. The QoL of each patient was assessed repeatedly over the study period.

**Fig. 1:**
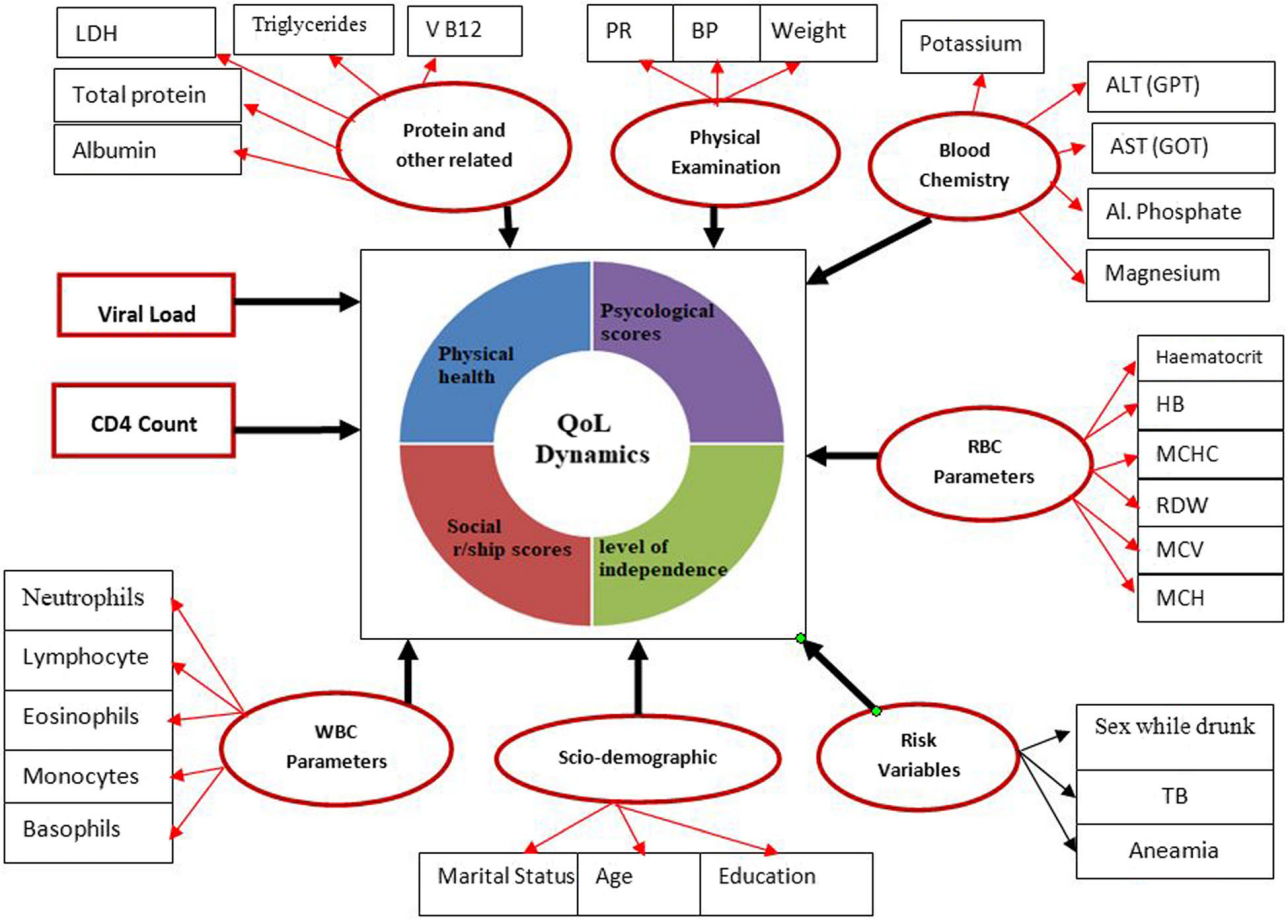
Graphical display of hypothesized model

The effect of several prognostic factors on long-term dynamics of QoL was investigated including (1) demographics: date of the clinical visit, age, gender, marital status, and educational status, (2) risk variables: sex under the influence of alcohol, contraceptive use, and substance use, (3) past opportunistic illness: tuberculosis and hypertension (4) clinical attributes: white blood cell parameters (lymphocyte count, neutrophil, leucocyte count, monocytes and eosinophils), RBC parameters (Hb, hematocrit, MCHC, MCV, MCH, RDW), physical examination parameters (blood pressures (BP), pulse rate (PR), weight and height), protein and other parameters, etc. (Fig. 1).

### Statistical methods

#### Factor analysis

Since our data have a large number of clinical variables, we used exploratory factor analysis in order to group and minimize the number of variables. Factor analysis was done by creating the principal components of the original variables. By using the Kaiser-criterions [30] and visual scree plot analysis [31], principal components (PC) with eigenvalues greater than 1 and PC that do not belong to the scree, were kept. A maximum likelihood extraction method with varimax rotation was used. Factor loadings describe the relationship of each clinical variable with each factor. The Factor loading is considered strong if it is greater than 0.6, moderate if it is in the range 0.4–0.6, and weak if less than 0.4 [32]. Each observation was assigned a score for each rotated factor on the basis of the loadings of the subject’s original variable levels. Accordingly, from the 24 clinical variables in the study, we managed to group them in order to create 9 PC, defined as granulocytes components, mononuclear components, eosinophils component, Hb and haematocrit component, red blood cell indices, liver abnormality component, electrolyte component, lipid component, and protein component. (See Table 1).

**Table 1:** Clinical parameters and corresponding factor loadings from the rotated factors

White blood cell parameters	Rotated Factors		
	1. Granulocytes component	2. Mononuclear component	3. Eosinophils component
Leucocyte	**0.925**	0.282	0.146
Neutrophils	**0.936**	−0.158	0.022
Lymphocytes	0.226	**0.838**	−0.109
Monocytes	**0.635**	0.417	−0.032
Eosinophils	0.085	0.058	**0.947**
Basophils	−0.035	**0.616**	0.339
Red Blood cell Parameters	Rotated Factors		
	1. Hb and Haematocrit component	2. Red blood cell indices component	
RBC counts	**0.946**	−0.130	
Hb	**0.886**	0.439	
Haematocrit	**0.919**	0.366	
MCV	0.075	**0.953**	
MCH	0.024	**0.825**	
MCHC	0.201	**0.521**	
RDW	−0.382	**−0.592**	
Blood Chemistry items	Rotated Factors		
	1. Liver enzyme abnormality component	2. Electrolyte component	
Chloride	−0.023	**0.455**	
Alkaline	0.174	0.032	
ALT (GPT)	**0.829**	−0.073	
AST (GOT)	**0.967**	−0.122	
Sodium	0.103	**0.994**	
Calcium	−0.020	**0.213**	
Protein and related	Rotated Factors		
	1. Lipid component	2. Protein component	
Cholesterol	**0.971**	0.027	
LDL	**0.917**	−0.129	
Triglycerides	**0.360**	0.341	
LDH	0.052	**−0.769**	
Total protein	−0.009	**0.670**	

### Multivariate multilevel model

The multilevel framework for medical data is important for both practical and methodological reasons. Practically, we are frequently interested in variability amongst advanced level factors, such as individual-to-individual variations. Methodologically, the multilevel framework leads to the relationship amongst the observations within a group. The multilevel framework can accommodate a cohort data and the correlation among observations [25, 33, 34]. However, the multilevel multivariate model, which extends the multilevel model to more than two response variables, has not been widely adopted within the medical and epidemiological studies [33, 35]. This work presented multivariate multilevel models for the long-term dynamics of QoL and illustrate how to interpret and fit models and compare the effects of covariates across the outcomes.

The separate multilevel models for each QoL domain score can be stated as:

1 $$ {y}_{kjt}=\kern0.5em {\boldsymbol{x}}_{\boldsymbol{tj}}^{\prime }{\boldsymbol{\beta}}_{\boldsymbol{k}}+{\boldsymbol{u}}_{\boldsymbol{k}\boldsymbol{j}}+{\boldsymbol{v}}_{\boldsymbol{k}\boldsymbol{j}}{T\mathrm{ime}}_{\boldsymbol{k}\boldsymbol{j}}+{e}_{kjt} $$

where *y*_*ktj*_ represents the k^th^ QoL domain scores at time *t* for patient *j.* The term ***u***_***kj***_ represents a random effect of patient-specific differences for the kth QoL domain score at baseline, while ***v***_***kj***_ represents a random effect of patient-specific differences for the kth QoL domain score in the rate of change and *e*_*kjt*_ represents random error. Since the data in this work are longitudinal cohort data, the observations for each individual may be correlated. The random-effects stated above to allow us to capture this correlation. Specifically, in Eq. (1) suppose that the QoL score variables are independent, and the random effects for each outcome are normally distributed. Assuming the residual errors for each of the outcomes are also normally distributed [25] it follows that,

2 $$ \left[\begin{array}{c}{u}_{kj}\\ {}{v}_{kj}\end{array}\right]\sim MVN\left(0,\left[\begin{array}{cc}{\sigma^2}_{u_k}& {\sigma}_{ukvk}\\ {}{\sigma}_{ukvk}& {\sigma^2}_{v_k}\end{array}\right]\ \right) $$

3 $$ {e}_{kj}\sim N\left(0,{\sigma^2}_{e_k}\right) $$

By fitting the above independent models in Eq. (1-3), we implicitly assume that the error terms are independent. However, the four domain scores from the same individuals are almost surely correlated. For example, the longitudinal change in QoL domain scores are more likely correlated with each other; thus, we can estimate the correlation amongst the random slopes. Moreover, because the QoL domain scores are repeated measures on the same individual, there will likely be a correlation between the residual errors [36]. A multivariate multilevel structure will address the above-mentioned limitations because it allows us to model the association between the outcome variables via correlations amongst the random effects and amongst the errors [33, 35].

In the separate analysis, we assume that the random effects are drawn from different four normal distributions. In a multilevel multivariate model, the random effects are drawn from a single multivariate normal distribution [37]. The specification of the covariance among random effects and the residual errors is as follow:

4 $$ \left[\begin{array}{c}\boldsymbol{U}\\ {}\boldsymbol{V}\end{array}\right]\sim \boldsymbol{MVN}\left(0,{\boldsymbol{\Omega}}_{\mathbf{G}}\right)\kern1em $$

5 $$ \boldsymbol{e}\sim \boldsymbol{MVN}\left(0,{\boldsymbol{\Omega}}_{\mathbf{R}}\right) $$

where **Ω**_**G**_ represents the variance-covariance matrix of all eight random effects and **Ω**_**R**_ represents the variance-covariance matrix of all four residuals. Furthermore, the multivariate multilevel framework allows us to estimate the variance-covariance matrix of patients intercepts and slope for each of the QoL domain scores. These variance-covariance structures can be interpreted substantively and give estimation improvement accuracy for the regression parameter and test power for the detection of covariates effect.

In this study, the normality assumption of longitudinal QoL domain scores was checked via the Q-Q plot using the transformed residuals errors based on the fitted model (as discussed by [38]). Secondly, we fit multivariate multilevel models for QoL outcome data. Thirdly, we considered the significant covariates effects for physical health, physiological score, level of independence and social relationship score in multivariate multilevel results. Finally, we compared the effects of covariates across the outcomes using a post-analysis contrasts.

All of the analyses were implemented using R-3.6.2. Statistical analysis was conducted assuming a two-sided 5% level of significance.

## Results

### Characteristics of cART cohort at enrolment

Table 2 shows the distribution of the socio-demographic and variables at the beginning of patient follow-up. All participants were black females (*n* = 219), with a median age of 25 years (Interquartile range, IQR, 22–30). The majority of participants were married or with a stable sexual partner 174 (80%), not co-infected with TB 201 (91.8%), not with anemia 208 (95.0%) and overweight or obese 137 (62.8%) based on their body mass index (BMI) measurements. Over half 153 (69.9%) reported having completed Grades 11 of schooling. With regard to the immunological state, 40.6 and 36.5% of patients an initial CD4 count of 350–499 and ≥ 500 cells/mm3, respectively. The log_10_ copies/ml VL count of the participants ranged from 1.47 to 6.81 with the first quartile of 3.84, a median of 4.46 and the third quartile of 5.06. (Table 2).

**Table 2:** Baseline characteristics of ART cohort in the CAPRISA 002 study

Variables	Count/Mean (Percentage/SD)
**Marital Status, n(%)**	
Single/no sexual partner	34 (15.5)
Married/stable sexual partner	174 (79.5)
Many sexual partners	11 (5.0)
**Educational Status, n(%)**	
Less than high school	16 (7.31)
High school	50 (22.8)
At least high school	153 (69.9)
**BMI Categories, n(%)**	
Underweight	5 (2.3)
Healthy weight	76 (34.9)
Overweight/Obese	137 (62.8)
**Immunologic state, n(%)**	
≥ 500 cells/mm3	80 (36.5)
350–500 cells/mm3	89 (40.6)
200–350 cells/mm3	41 (18.7)
≤ 200 cells/mm3	9 (4.1)
**TB Co-infected, n(%)**	
Yes	18 (9.2)
No	201 (91.8)
**Anemia, n(%)**	
Yes	11 (5.0)
No	208 (95.0)
**Contraceptive use, n(%)**	
Yes	179 (81.7)
No	40 (18.3)
**Sex act under the influence of alcohol, n(%)**	
Yes	22 (10.0)
No	197 (90.0)
**Baseline age, Median (IQR)**	25.0 (22.0–30.0)
**Baseline log viral load, Median (IQR)**	4.46 (3.84–5.06)

### Longitudinal change of QoL scores of HIV-infected patients

**Fig. 2:**
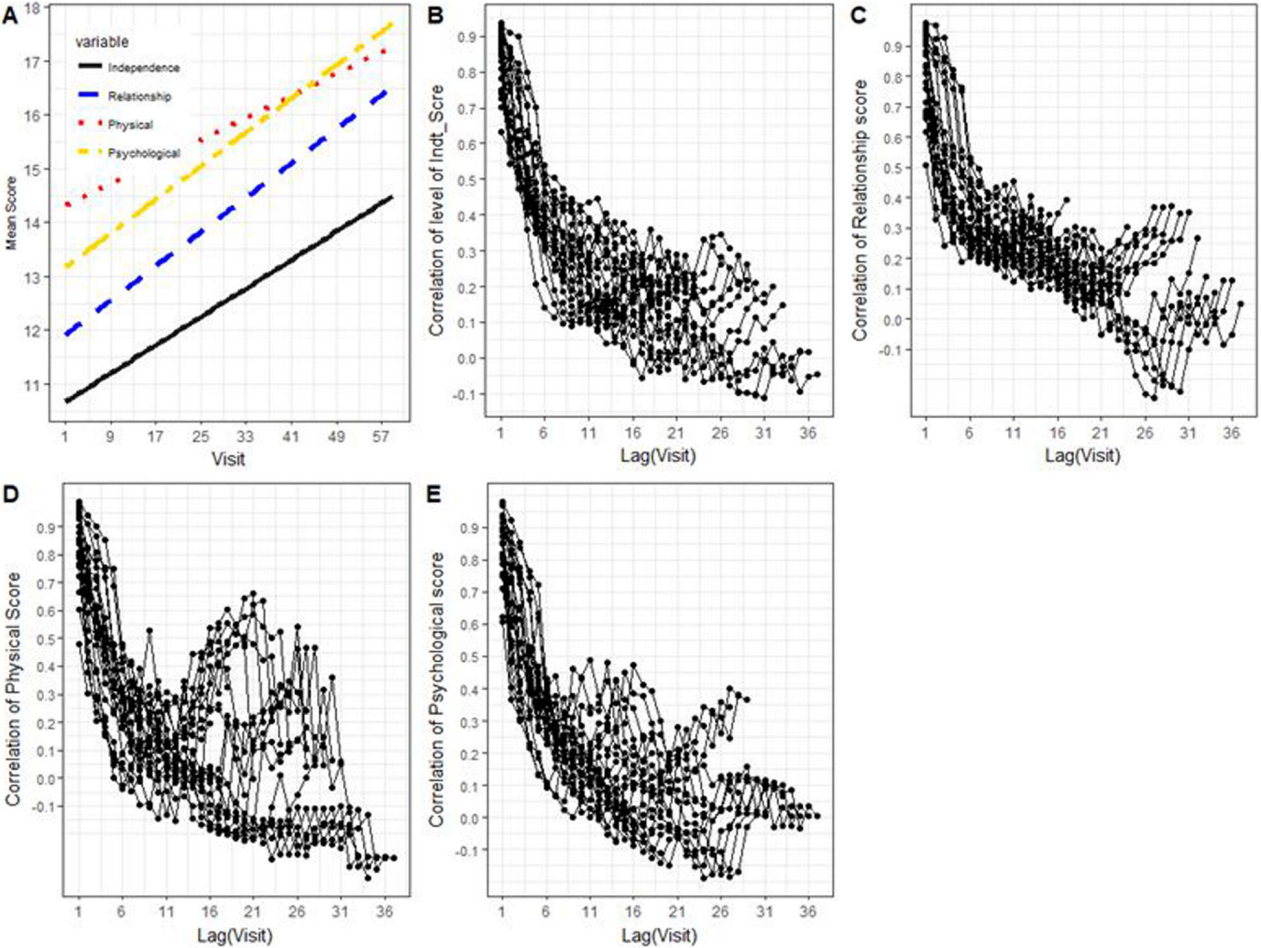
**a** Smoothed line plots of HRQOL scores, **b**-**e** Correlogram of level of Independences, social relationship scores, physical health and physiological scores respectively

**Fig. 3:**
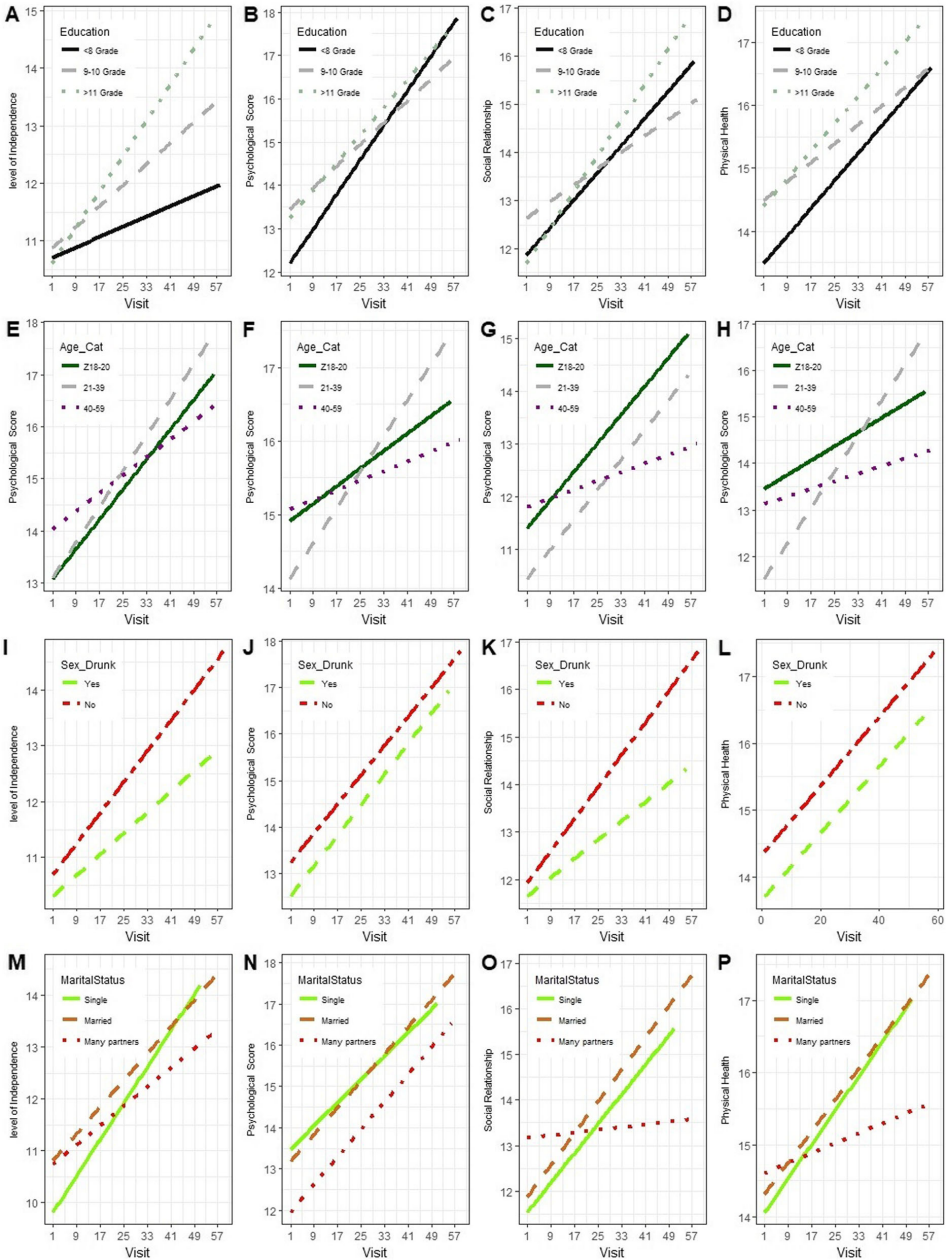
Trends of QoL scores over time based on socio-demographic variables

The line plot in Fig. 2a, displays the trends of QoL of all HIV patients, over time. From these plots, we can see that the overall QoL scores among HIV-infected patients increased throughout the duration of follow-up periods. The longer a patient stayed under follow up, the more she experienced an increase of her QoL scores. From Fig. 3(a-d), we note that the overall long time trends of QoL scores of patients with higher education levels had increased with greater rates as compared to those patients with lower education (< 8 grade). The trend of QoL (mainly physical health and social relationship) of younger and older adult women increased at slower rates compared to those who were middle-aged (Fig. 3e-h).

**Fig. 4:**
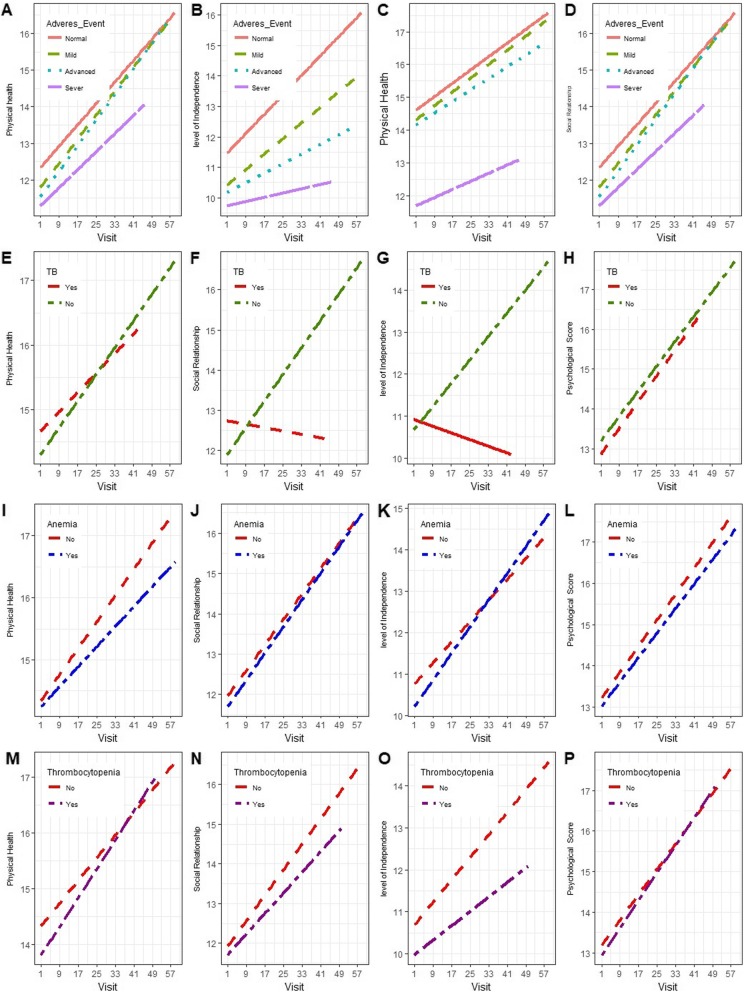
Trends of QoL scores over time based on clinical factors

Long term QoL trends were evaluated for patients with different categories of clinical factors. Figure 4(a-d) depicts, increased QoL scores rate of change over time for patients with higher CD4 cell count levels as compared to those patients with lower CD4 cell counts. Figure 4(e-h) shows that the trends of QoL scores increased with greater rates for patients without TB as compared to those who were TB co-infected.

### Examining the relationship between QoL domain scores

The multivariate multilevel framework allows us to estimate the correlation matrix between the patient’s slopes for each of the QOL domain scores (Table 3). The correlation among the intercepts was 0.94, 0.61, 0.60 and 0.58, showing a strong positive correlation between baseline values for physical health and psychological score, physical health and social-relationship, level of independence and social-relationship, and social-relationship and psychological score, respectively. Furthermore, the correlation amongst the slopes was 0.90, 0.86, 0.84, 0.84, 0.81 and 0.81, showing a strong positive correlation between rates of change (slope) values of domain scores with time.

**Table 3:** Estimated correlation matrix of the multivariate multilevel model

	baseline level of independence	baseline physical health	baseline psychological	baseline social r/ship	Time Slope level of independence	Time slope physical	Time slope psychological
baseline level of independence 1							
baseline physical health	0.49	1					
baseline Psychological	0.42	0.94	1				
baseline Social R/ship	0.60	0.61	0.58	1			
Time Slope level of Independence	−0.60	− 0.66	− 0.53	− 0.51	1		
Time slope physical	−0.50	− 0.93	− 0.84	− 0.60	0.84	1	
Time slope psychological	−0.47	− 0.93	− 0.89	− 0.58	0.81	0.90	1
Time slope social r/ship	−0.48	− 0.65	− 0.56	− 0.69	0.86	0.84	0.81

Table 3 also showed that there is a strong negative relationship between baseline values of physical health and slope value of physical health, baseline values for psychological score and slope value of psychological score, baseline values for level of independence and slope value of level of independence score and baseline values for social relationship and slope value of social relationship domain score. The correlations between different parameters across QoL domain scores were − 0.93, − 0.65 and − 0.61, indicating a strong negative relationship between the baseline of physical health domain scores and the rate of change in psychological score, level of independence and social relationship, respectively. There was thus a relationship between a patient’s baseline position on one QoL domain and the rate of change on the other patient-specific.

### Multivariate multilevel model results

Table 4 presents the fit statistics for the independent outcome and multivariate (related outcome) model for QoL domain scores of HIV infected patients. Both models include a linear effect of time (month) and the covariates. However, comparing the log-likelihood and AIC values shows that the multivariate multilevel structure was significantly better than separate multilevel models and this implies that there is a strong association among the four markers. Furthermore, model diagnostics have been performed and the residual and influence diagnostics affirmed no violation of implicit and explicit assumptions in our model.

**Table 4:** Fit statistics for the independent outcome and multivariate (related outcome) analysis

Criteria	Independent Outcome	Related Outcome
−2 Log Likelihood	39,811.9	36,111.0
AIC	40,201.9	36,587.0
AICC	40,210.9	36,600.4
BIC	40,862.8	37,393.6

The results of the multivariate multilevel model are presented in Table 5. Patients with normal disease stages were significantly associated with a higher baseline physical health (β = 3.33; 95%CI:2.11, 4.55) and psychological scores (β = 1.43; 95%CI:1.13, 3.18) as compared to those with severe diseasing stage. Similarly, patients with mild disease stage were significantly associated with a higher baseline physical health and psychological score as compared to those patients with severe diseasing stage. Furthermore, patients with thrombocytopenia were significantly associated with a smaller baseline level of independence. Patients without anemia and TB co-infection had tended to have a better average baseline level of independence as compared to those with anemia and TB co-infection.

**Table 5:** Results of a multivariate multilevel model for QOL domain scores

Effects	Physical Health	Psychological Score	Level of Independence	Social Relationship
Intercepts	10.8 (7.43, 14.2)**	9.63 (6.28, 12.9)**	12.8 (8.7, 16.98)**	13.5 (9.9, 17.16)*
Time	0.18 (0.01, 0.37)**	0.21 (0.04, 0.38)**	0.05(− 0.27, 0.18)	0.03(− 0.19, 0.14)
Thrombocytopenia: Yes	0.67(− 0.56, 1.89)	0.52(− 0.69, 1.73)	− 2.01(−3.89, − 0.13)*	− 0.33(−1.93, 1.27)
TB: No	0.38 (0.02, 0.74)**	0.01(− 0.35, 0.38)	0.16(− 0.48, 0.80)	0.05(− 0.47, 0.56)
Anemia: No	1.38 (1.17, 2.12)*	− 0.61(− 1.37, 0.15)	−0.58(− 1.35, 0.18)	0.06(− 0.96, 1.09)
Adverse Event: Normal	3.33 (2.11, 4.55)***	1.43 (1.23, 3.18)*	1.64 (0.42, 2.87)	1.64 (1.02, 2.87)*
Adverse Event: Mild	2.86 (1.66, 4.06)*	1.23 (1.03, 2.43)***	−0.08(− 1.98, 1.82)	1.44 (0.18, 3.06)
Adverse Event: Advanced	3.34 (2.16, 4.53)*	1.32 (0.14, 2.51)	− 0.76(− 2.65, 1.13)	0.86(− 0.76, 2.47)
Education: Grade 9–10	0.60 (0.26, 0.88)	0.25(− 0.60, 1.09)	0.53(− 0.41, 1.48)	0.65(− 0.20, 1.50)
Education:> Grade 11 Grade	0.22 (0.01, 0.99)*	1.26 (1.18, 2.89)**	0.54(− 0.79, 1.86)	0.20(− 0.99, 1.39)
Marital Status: Single/no sexual partner	0.20(−1.43, 1.83)	1.25 (0.38, 2.89)	− 0.67(− 2.45, 1.11)	− 0.51(− 2.10, 1.07)
Marital Status: Married (stable sexual partner)	0.13(−1.69, 1.94)	2.14 (1.01, 3.87)*	−1.36(− 3.34, 0.62)	−1.10(− 2.87, 0.67)
Age: 21–39 years	− 0.34(− 1.16, − 0.49)*	0.26(− 0.59, 1.12)	−1.89(− 2.97, − 0.81)*	−1.25(− 2.19, − 0.30)*
Age: 40–59 years	0.26(− 1.30, 1.81)	0.79(− 0.82, 2.39)	−1.49(− 3.48, 0.49)	− 0.09(− 1.84, 1.66)
Sex while drunk: Yes	−0.08(− 0.99, 0.82)	0.22(− 0.72, 1.16)	0.03(− 1.18, 1.24)	− 0.49(− 1.55, 0.57)
Weight	0.01 (0.001, 0.03)*	0.01 (0.002, 0.03)*	0.01(− 0.01, 0.03)	− 0.01(− 0.02, 0.01)
Viral Load	**−0.21(− 0.39, − 0.03)**	−0.29(− 0.46, − 0.10)*	−0.09(− 0.37, − 0.01)*	−0.06(− 0.30, − 0.01)*
RBC indices	0.15(− 0.09, 0.38)	0.28 (0.04, 0.51)*	− 0.22(− 0.56, 0.13)	0.04(− 0.25, 0.34)
Hb and haematocrit component	−0.04(− 0.26, 0.18)	−0.11(− 0.33, 0.12)	−0.18(− 0.51, 0.15)	0.05(− 0.24, 0.33)
Granulocytes component	−0.05(− 0.23, 0.13)	0.06(− 0.12, 0.25)	− 0.06(− 0.33, 0.21)	0.14(− 0.09, 0.37)
Mononuclear component	0.28 (0.09, 0.48)*	− 0.13(− 0.30, 0.05)	0.21(− 0.05, 0.47)	0.10(− 0.12, 0.32)
Eosinophil component	− 0.04(− 0.12, 0.03)	− 0.07(− 0.15, 0.00)	−0.12(− 0.24, 0.01)	0.04(− 0.07, 0.15)
Liver enzymes abnormality component	−0.08(− 0.04, − 0.08)*	−0.18(− 0.43, − 0.09)*	0.14(−0.12, 0.40)	−0.15(− 0.37, 0.07)
Electrolyte component	−0.06(− 0.22, 0.10)	−0.18(− 0.34, 0.02)	0.28 (0.02, 0.53)*	−0.10(− 0.32, 0.11)
**Time Slope**				
Thrombocytopenia: Yes	−0.03(− 0.08, 0.01)	−0.02(− 0.06, 0.02)	−0.16 (− 0.23,-0.08)*	0.02(− 0.04, 0.08)
Viral Load	−0.06(− 0.1, − 0.001)**	−0.01(− 0.09, − 0.002)*	−0.001(− 0.01, 0.01)	−0.001(− 0.01, 0.01)
Electrolyte component	0.01 (0.001, 0.02)*	0.04 (0.01, 0.06)*	0.00(− 0.01, 0.01)	0.00 (0.00, 0.01)
RBC indices	0.00(−0.01, 0.01)	−0.01(− 0.02, 0.00)	0.01 (0.00, 0.03)	0.00(− 0.01, 0.01)
Hb and haematocrit component	0.06 (0.01, 0.09)*	0.02 (0.01, 0.03)*	0.02 (0.01, 0.03)*	0.01 (0.001, 0.02)*
Granulocytes component	0.00 (0.00, 0.01)	0.00(− 0.01, 0.00)	0.01 (0.00, 0.02)	0.00(− 0.01, 0.01)
Mononuclear component	0.001(− 0.01, 0.01)	− 0.01(− 0.02, 0.001)	0.02 (0.01, 0.02)*	0.001(− 0.01, 0.002)
Liver enzymes abnormality component	− 0.01(− 0.02, − 0.001)*	−0.01(− 0.02, 0.001)	−0.01(− 0.02, 0.002)	−0.01(− 0.02, 0.001)
Weight	0.01 (0.001, 0.02)*	0.01 (0.001, 0.02)*	0.001(− 0.01, 0.01)	0.001(− 0.01, 0.01)
Adverse Event: Normal	0.03 (0.01, 0.05)**	− 0.03(− 0.11, 0.04)	0.09 (0.01, 0.17)**	−0.04(− 0.12, 0.04)
Adverse Event: Mild	0.01 (0.008, 0.06)**	−0.03(− 0.11, 0.04)	0.06 (0.03, 0.12)**	−0.04(− 0.12, 0.04)
Adverse Event: Advanced	−0.04(− 0.12, 0.03)	−0.04(− 0.11, 0.04)	0.08 (0.00, 0.16)**	−0.02(− 0.10, 0.06)
Anemia: No	0.03 (0.001, 0.07)**	0.02(− 0.01, 0.06)	−0.06(− 0.11, 0.01)	0.001(− 0.04, 0.04)
Education: Grade 9–10	0.001(− 0.02, 0.02)	0.001(− 0.02, 0.02)	0.04 (0.01, 0.08)***	−0.02(− 0.04, 0.00)
Education:> Grade 11	−0.01(− 0.04, 0.03)	0.01(− 0.02, 0.04)	0.03 (0.01, 0.09)*	0.01(− 0.03, 0.04)
Marital Status: Single	0.01(− 0.02, 0.04)	0.00(− 0.04, 0.03)	−0.02(− 0.06, 0.01)	0.03(− 0.01, 0.07)
Marital Status: Married (stable sexual partner)	−0.01(− 0.05, 0.03)	−0.02(− 0.06, 0.02)	−0.01(− 0.06, 0.03)	0.05 (0.01, 0.10)**
Age: 21–39 years	0.02 (0.01, 0.04)*	0.00(−0.03, 0.04)	0.07 (0.03, 0.12)*	0.06 (0.02, 0.09)*
Age: 40–59 years	−0.01(− 0.07, 0.06)	−0.01(− 0.07, 0.06)	0.07(− 0.01, 0.16)	0.01(−0.05, 0.08)
Sex while Drunk: Yes	− 0.01(− 0.03, 0.01)	−0.01(− 0.03, 0.01)	−0.06(− 0.08, − 0.04)*	−0.03(− 0.06, − 0.01)*

**Key**:- Statistical significance: (*)*P* < 0.05; (**)*P* < 0.01; (***)*P* < 0.001; Reference category: Age [≤20]; education [≤8 grade]; marital status [many sexual partners]; Sex while Drunk [No]; TB [Yes]; Adverse event [Severe] and Thrombocytopenia [No]

Patients with higher education levels tended to have a better baseline physical health scores (β = 0.22; 95%CI:0.01–0.99) and psychological scores (β = 1.26; 95%CI:1.18, 2.89), as compared to those patients with lower education. Patients who reported stable sexual partners (β = 2.14; 95%CI:1.01, 3.87) were significantly associated with higher baseline psychological scores as compared to those who report many sexual partners. The result further showed that patients within an age group 21–39 had a smaller baseline physical health, level of independence and social relationship scores as compared to those patients within age group ≤20. Moreover, increased weight is associated with increased baseline physical health score and psychological score. With regard to clinical parameters, we noted that patients having high liver enzyme abnormality were significantly associated with lower baseline physical health and psychological scores. The result further showed that an increased mononuclear and electrolytes components are associated with increased physical health and level of independence, respectively.

We noted that an increase Hb and haematocrit component increases the rate of change of physical health (β = 0.02; 95%CI:0.01, 0.03), psychological scores (β = 0.02; 95%CI:0.01, 0.03), level of independence (β = 0.02; 95%CI:0.01, 0.03) and social relationship (β = 0.01; 95%CI:0.001, 0.02) through the follow-up time. As the latent variable related to electrolytes increases, the rate of change in physical health scores increases with time. The results further showed that patients with thrombocytopenia had a smaller rate of change of level of independence score as compared to those without thrombocytopenia. Patients with normal and mild disease stage tended to have a better rate of change of physical health score and level of independence score through the follow-up time. Moreover, an increase in viral load decreases the average rate of change in physical health score and psychological score. Similarly, an increase in liver enzyme abnormality decreases the rate of change in physical health through the follow-up time.

Patients with higher education levels tended to have a better rate of change of level of independence score with time, as compared to those with a low level of education (< 8 grade). An increase in weight increases the rate of change in physical health and psychological scores through the follow-up time. Patients within the age group 21–39, tended to have a better rate of change of physical health, level of independence and social relationship scores as compared to those patients with age ≤ 20. Moreover, patients having sex whilst under the influence of alcohol had a smaller rate of change of level of independence and social relationship scores with time, as compared to those who don’t have sex whilst under the influence of alcohol.

### Comparing the effects of covariates across the outcomes

Table 6 presents the results of the effect of covariates across the outcomes. We considered the significant covariate effects for physical health, physiological score, level of independence and social relationship score in Table 5. CD4 count (normal versus severe) has a differential effect across categories but, in general, the direction of the effect is the same for all “baseline physical health score”, “baseline social relationship scores” and “baseline psychological scores”. Similarly, the effects of the mild diseasing stage are significantly larger for baseline physical health score than for baseline social relationship scores. The results further showed that education (higher education versus lower education), age (21–39 age category versus < 21 age category) and viral load have a significant differential effect across QoL domain scores.

**Table 6:** Results from comparing the effects of covariates across the outcomes

Effects	Label	Estimate	Standard Error	P-value
Adverse Event: Normal	Physical Health - Physiological score	1.91	0.196	0.002**
Adverse Event: Normal	Physical Health - Social r/ship score	1.69	0.426	0.022*
Adverse Event: Mild	Physical Health - Physiological score	1.64	0.089	0.000***
Adverse Event: Advance	Physical Health - Physiological score	2.02	0.195	0.000***
Age: 21–39	Physical Health - Level of independence	1.55	0.027	0.000***
Age: 21–39	Physical Health - Social r/ship score	0.91	0.097	0.004**
Education:> 11 grade	Physical Health - Physiological score	−1.04	0.197	0.001**
Weight	Physical Health - Physiological score	0.001	0.002	0.688
Viral Load	Level of independence - Social r/ship	−0.03	0.16	0.839
Viral Load	Physical Health- Social r/ship score	−0.15	0.07	0.042*
Viral Load	Physiological score- Social r/ship score	−0.23	0.09	0.031*
Liver Abnormality	Physical Health - Physiological score	0.1	0.07	0.415
**Time Slope**				
Viral Load	Physical Health - Physiological score	−0.05	0.01	0.017*
Age: 21–39	Level of independence - Physical Health	−0.06	0.01	0.011*
Age: 21–39	Social r/ship - Physical Health	−0.05	0.02	0.041*
Hb and haematocrit component	Level of independence - Social r/ship	0.01	0.06	0.362
Hb and haematocrit component	Physical Health- Social r/ship	0.06	0.02	0.042*
Hb and haematocrit component	Physiological score- Social r/ship	0.01	0.05	0.553
Weight	Physical Health - Physiological score	−0.01	0.02	0.491
Electrolyte Components	Physical Health - Physiological score	−0.04	0.03	0.101
Normal adverse event	Physical Health - Level of independence	−0.05	0.04	0.138
Mild adverse event	Physical Health - Level of independence	−0.04	0.06	0.699
Sex while drunk	Level of independence - Social r/ship	−0.03	0.06	0.948

**Key**:- Statistical significance: (*)*P* < 0.05; (**)*P* < 0.01; (***)*P* < 0.001

The effect of time slope covariates across the outcomes were also compared. As seen from Table 6, we noted that the effects of viral load are significantly larger for the rate of change of physical health score than for psychological scores. Likewise, the effects of Hb and haematocrit are significantly larger for the rate of change of physical health score and psychological scores than for the rate of change of social relationship score. Moreover, age (21–39 age category versus < 21 age category) have a significant differential effect across QoL domain scores.

## Discussion

In this paper, we presented the multivariate multilevel model which accounts for both the multivariate nature of the outcome and the hierarchical structure of the data. A multivariate multilevel model offers advantages over a separate model for each outcome. The multivariate multilevel approach allows us to gain clinically meaningful adjusted association parameters and more efficient parameter estimates. Moreover, it provides the correlations among the outcomes at the baseline and follow-up time levels. As Tate and Pituch [39] also noted, the multivariate multilevel model provides more accurate standard errors and powerful tests of the covariates as compared to a separate modelling approach. Griffiths et al. [23] also showed that the multivariate modelling assumptions are not violated and the parameter estimates are stable, which we did not find with the separate modelling approach. Furthermore, as noted [39], the multivariate multilevel approach allows the researchers to take into account any missing data for the outcome variables, eliminating the need to delete observations in separate analyses. A further advantage of the multivariate multilevel model is that it facilitates tests of the equivalence of covariate effects across the outcome variables and could also be used to test the effect of covariates at different time points in a longitudinal design. However, since the dimension of the random effects is often high and the density functions of the multivariate multilevel model can be highly complicated, evaluation of the likelihood (integral) can be a major challenge and very intensive.

Our results showed that patients with higher educational level had a significant effect on the overall long-term trends of QoL scores of patients. Tomita et al. [40], Gaspar et al. [41] and Belak Kovačević et al. [42] also found that higher education promotes better QoL, possibly due to better knowledge about their treatment and disease, access to health services, or functional status.

QoL scores (mainly physical health and level of independence score) of patients with higher CD4 cell count levels, increased at a greater rate as compared to those with lower CD4 cell counts. These findings are in line with findings reported in previous studies [22, 40, 43–46], where it was reported that increased CD4 cell count over time, led to better QoL outcomes. The trends of QoL scores of patients without TB, increased with greater rates as compared to those who were co-infected, a finding that is in accordance with previously reported research [47], who found that co-infected patients had a lower QoL scores as compared to those living with HIV without TB. Hemoglobin abnormality was also a factor of long-term QoL trend scores of patients, a finding that is in accordance with previous reports [48], where it was found that an increase of hemoglobin level was associated with greater improvement in QoL scores. Patients without thrombocytopenia, was associated with a better level of independence. Similarly, higher scores of latent variables related to electrolytes latent and RBC parameters, lower scores of latent variables related to liver abnormality and lower viral loads significantly improve the rate of change of QoL (mainly physical health score) HIV infected patients.

This study has some limitations, including the missing data, which are expected for a long term follow-up study conducted on data collected from patients’ files involving many variables. Another limitation of this study is that we did not include religion domains scores to our model because more than 40% of observations for those domains score are missing. Moreover, some socio-demographic variables that influence long term QoL trends, may not have been included, for example, cites (rural/urban cites). Furthermore, the study findings were limited to adult females.

## Conclusions

Overall, from a methodological perspective, this article presents applications for the analysis of hierarchical multivariate response data. As we have shown, these hierarchical multivariate assumptions can be addressed with a multivariate multilevel model in ways that are not possible with univariate (separate) models. This approach provides wide-ranging information about the overall QoL for HIV patients. Though this research presented the usefulness of the multivariate multilevel model for analyzing HIV-infected patients QoL, the approach is applicable to a wide variety of chronic diseases. There is a need for increased research in terms of methods, so hopefully, this article will be helpful to applied researchers (for medical research) to familiarize with the method and interpretation of the results therefrom. From a clinical perspective, modelling of long-term QoL of HIV-infected patients is very useful for improving patient outcomes and formulating an ART management plan.

## Data Availability

The study data is available upon request.

## Abbreviations

PLHIV: People living with HIV
HB: Hemoglobin
V B12: Vitamin B12
BP: Blood pressure
LDH: Lactate dehydrogenase
PR: Pulse rate
RBC: Red blood cells
QoL: Quality of life
CAPRISA: Center of the AIDS programme of research in South Africa
OI: *Opportunistic infections*
*ARV*: Antiretroviral (drug)
ART: Antiretroviral therapy
*AIDS*: Acquired Immune Deficiency Syndrome
RDW: Red cell distribution width
HIV: Human Immunodeficiency Virus
MCH: Mean corpuscular hemoglobin
MCV: Mean corpuscular volume
MCHC: Mean corpuscular hemoglobin concentration
AST: Aspartate aminotransferase
ALT: Alanine aminotransferase
